# Algal endosymbionts as vectors of horizontal gene transfer in photosynthetic eukaryotes

**DOI:** 10.3389/fpls.2013.00366

**Published:** 2013-09-19

**Authors:** Huan Qiu, Hwan Su Yoon, Debashish Bhattacharya

**Affiliations:** ^1^Department of Ecology, Evolution, and Natural Resources, Institute of Marine and Coastal Science, Rutgers UniversityNew Brunswick, NJ, USA; ^2^Department of Biological Sciences, Sungkyunkwan UniversitySuwon, South Korea

**Keywords:** algal evolution, Plantae plastid origin, primary endosymbiosis, chromalveolates, EGT, HGT

## Abstract

Photosynthesis in eukaryotes occurs in the plastid, an organelle that is derived from a single cyanobacterial primary endosymbiosis in the common ancestor of the supergroup Plantae (or Archaeplastida) that includes green, red, and glaucophyte algae and plants. However a variety of other phytoplankton such as the chlorophyll *c*-containing diatoms, dinoflagellates, and haptophytes contain a red alga-derived plastid that traces its origin to secondary or tertiary (eukaryote engulfs eukaryote) endosymbiosis. The hypothesis of Plantae monophyly has only recently been substantiated, however the extent and role of endosymbiotic and horizontal gene transfer (EGT and HGT) in algal genome evolution still remain to be fully understood. What is becoming clear from analysis of complete genome data is that algal gene complements can no longer be considered essentially eukaryotic in provenance; i.e., with the expected addition of several hundred cyanobacterial genes derived from EGT and a similar number derived from the mitochondrial ancestor. For example, we now know that foreign cells such as Chlamydiae and other prokaryotes have made significant contributions to plastid functions in Plantae. Perhaps more surprising is the recent finding of extensive bacterium-derived HGT in the nuclear genome of the unicellular red alga *Porphyridium purpureum* that does not relate to plastid functions. These non-endosymbiont gene transfers not only shaped the evolutionary history of Plantae but also were propagated *via* secondary endosymbiosis to a multitude of other phytoplankton. Here we discuss the idea that Plantae (in particular red algae) are one of the major players in eukaryote genome evolution by virtue of their ability to act as “sinks” and “sources” of foreign genes through HGT and endosymbiosis, respectively. This hypothesis recognizes the often under-appreciated Rhodophyta as major sources of genetic novelty among photosynthetic eukaryotes.

## INTRODUCTION

**FIGURE 1:**
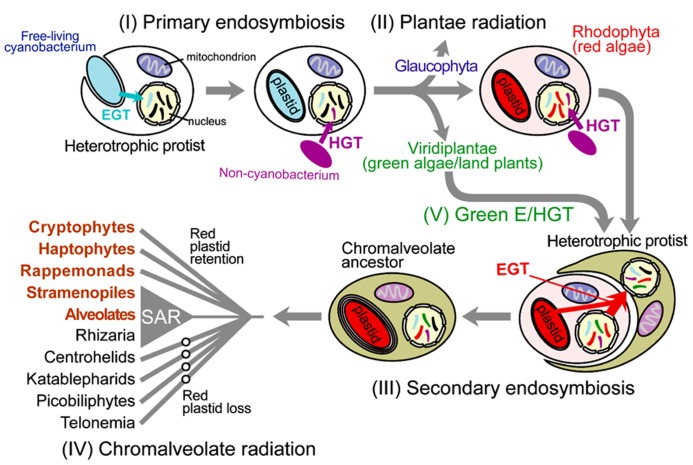
**Schematic illustration of cyanobacterial primary endosymbiosis and red algal secondary endosymbiosis that gave rise to the plastid in the vast majority of photosynthetic eukaryotes.** Gene movement *via* endosymbiotic gene transfer (EGT) and horizontal gene transfer (HGT) is indicated with the arrows. Important intracellular organelles (i.e., nucleus, mitochondrion, and plastid) are shown. Genetic material of non-lineal evolutionary origin in the nucleus is presented as stripes of different colors with the color indicating the source of the gene.

Photosynthetic eukaryotes (i.e., algae and plants) are a taxonomically diverse group with a wide variety of cell morphologies (e.g., diatoms, dinoflagellates, coccolithophores) and lifestyles that are key primary producers (Field et al., 1998). All eukaryotic photosynthesis relies on the intracellular organelle, the plastid (chloroplast in plants and green algae) that was derived over one billion years ago from a cyanobacterial primary endosymbiosis. In this process, a once free-living cyanobacterium capable of oxygenic photosynthesis was engulfed and retained in a heterotrophic protist, and over time evolved into the intracellular organelle (Section I in **Figure 1**; Cavalier-Smith, 1999; Bhattacharya et al., 2004). The resulting plastid-harboring protist ancestor gave rise to three lineages of Plantae (or Archaeplastida); i.e., Glaucophyta, Rhodophyta (red algae), and Viridiplantae (green algae and land plants; Section II in **Figure 1**; Adl et al., 2005). The establishment of Plantae plastid monophyly (e.g., Rodriguez-Ezpeleta et al., 2005) and, only recently, the monophyly of Plantae hosts (Chan et al., 2011; Price et al., 2012) provides strong support for the idea that the Plantae primary endosymbiosis occurred once in evolution. Despite its groundbreaking impact on eukaryote evolution and overall, the trajectory of life on Earth, primary endosymbiosis appears to be exceedingly rare. The only other known case of plastid primary endosymbiosis is provided by a single lineage of Rhizaria, *Paulinella* (Lauterborn, 1895; Yoon et al., 2006), which acquired a *Synechococcus*-like alpha-cyanobacterium ~65 million years ago (Nowack et al., 2008). The rarity of primary endosymbiosis is ascribed to difficulties in the initial “domestication” of the wild-type cyanobacterium and its integration into host cell metabolism. It is believed that primary endosymbiosis in the Plantae ancestor was made possible by the concomitant infection by parasitic Chlamydiae (Huang and Gogarten, 2007). Recent work suggests that effector proteins secreted by Chlamydiae might have facilitated the integration of carbon metabolism between the cyanobacterial endosymbiont and the host (Ball et al., 2013; Baum, 2013).

Whereas eukaryotic photosynthesis commenced with primary endosymbiosis, its greatest impact was achieved through additional rounds of secondary and tertiary endosymbiosis, whereby the cyanobacterium-derived organelle was transferred to a myriad of other protist hosts (e.g., red algal endosymbiosis; Section III in **Figure 1**; Keeling, 2010; Dorrell and Smith, 2011). Green algae were taken up at least three times by the ancestors of chlorarachniophytes, euglenids, and some “green” dinoflagellates (Archibald and Keeling, 2002; Rogers et al., 2007; Dorrell and Smith, 2011). The red algal plastid is found in diverse taxa such as cryptomonads, haptophytes, heterokonts, dinoflagellates, and apicomplexans, which collectively are often referred to as “chromalveolates” due to the presence of chlorophyll *c* in many of their plastids (Cavalier-Smith, 1999). Whether chromalveolates constitute a monophyletic group (Lane and Archibald, 2008; Keeling, 2009), however, clearly not under the scheme envisioned by (Cavalier-Smith, 1999), and whether the red alga-derived plastid found in many of its constituent taxa are derived from a single red algal endosymbiosis event (Section IV in **Figure 1**; Keeling, 2010) remain subjects of active debate. Even more complicated is tertiary endosymbiosis, in which secondary plastid-containing algae were engulfed and reduced to endosymbionts. This process has occurred multiple times in dinoflagellate lineages (Keeling, 2010) as evidenced by the haptophyte-derived plastid in *Karenia* and *Karlodinium* spp. (Hansen et al., 2000), the diatom-derived plastid in taxa such as *Kryptoperidinium foliaceum* (Chesnick et al., 1997), and the cryptophyte-derived plastid in *Dinophysis* spp. (Chesnick et al., 1996; Park et al., 2010; Kim et al., 2012).

In addition to the clear instances of plastid endosymbiosis described above in which the organelle is retained in the cell and identifies the donor, are the other more intriguing cases of plastid replacement. When these events are recent and the ancestral plastid source is unambiguous, then the inference is trivial even when both plastid sources are ultimately of the same origin (e.g., dinoflagellate peridinin-containing “red” plastid is replaced by a haptophyte “red” plastid; Ishida and Green, 2002). Apart from phylogenetic signal embedded in the organelle genome, “footprints” of the two endosymbionts can also be found in the nuclear genome in the form of transferred genes associated with each event (Nosenko et al., 2006). However if the cryptic endosymbiosis occurred in deep time (e.g., hundreds of millions of years ago), then such a hypothesis is exceedingly difficult to test if the plastid donors derive from the same ancestral lineage; i.e., making it intractable to discriminate between genes associated with each event. However if the plastid donors are phylogenetically distantly related then it may be possible to identify cases of cryptic endosymbiosis. We proposed such a case involving a cryptic green algal endosymbiosis, initially described in diatom genomes, and then more broadly applied to chromalveolates (Section V in **Figure 1**; Moustafa et al., 2009). Under this scenario, the cryptic green alga-derived plastid was presumably replaced by the canonical red algal endosymbiont in these taxa. An opposite case is found in the chlorarachniophyte *Bigelowiella natans*, which contains a green alga-derived secondary plastid but encodes a large number of nuclear-encoded genes of red algal origin (Curtis et al., 2012), potentially derived from the ancient red algal endosymbiont shared by the common ancestor of rhizarians and chromalveolates. Regardless of their mechanism of origin, it is now clear that chromalveolates and rhizarians share a large number of genes of both red and green algal origin. Compared to primary endosymbiosis, once “eukaryotization” of a plastid endosymbiont has occurred then its transfer is more likely. This sort of eukaryote-to-eukaryote plastid transfer resulted in a great deal of plastid diversity and to a large assemblage of taxa with significant ecological, economic, and health significance than the Plantae lineages alone (Simon et al., 2009; Keeling, 2010).

All photosynthetic eukaryotes have undergone extensive foreign gene transfer (Keeling and Palmer, 2008), particularly from the plastid donor *via* endosymbiotic gene transfer (EGT; **Figure 1**; Timmis et al., 2004). In addition to receiving genes from the endosymbiont, algae and plants also acquire foreign genes from non-cyanobacterial prokaryotes *via* horizontal gene transfer (HGT; **Figure 1**). In contrast to vertical genetic inheritance from parent to offspring, HGT is the genetic movement across species without the involvement of reproduction (Doolittle, 1999). Whereas HGT has long been known as a major force in prokaryote evolution (Gogarten et al., 2002; Boucher et al., 2003), its significance to eukaryote evolution has only recently been appreciated (Keeling and Palmer, 2008; Andersson, 2009; Dunning Hotopp, 2011; Bhattacharya et al., 2013; Wijayawardena et al., 2013). At the broadest level, endosymbiotic (E)/HGT can be thought of as a pipeline that allows the flow of genetic information across branches in the tree of life. Below we summarize recent studies of E/HGT in algae and plants. In particular we focus on complete genome data that was recently generated from the mesophilic, unicellular red alga *Porphyridium purpureum* (Bhattacharya et al., 2013). We determine the significance of E/HGT in this species from prokaryote sources, and elucidate the role of red algae as mediators of prokaryotic gene spread among taxa that contain a red alga-derived plastid.

## ENDOSYMBIOTIC/HORIZONTAL GENE TRANSFER OF PROKARYOTIC GENES IN PLANTAE

In the process of plastid origin, the endosymbiont undergoes dramatic genome reduction leading to highly reduced modern-day plastid genomes encoding <250 genes. This genome reduction is explained in part by the movement of hundreds of cyanobacterium-derived genes to the host nuclear genome *via* EGT (**Figure 1**). Many of the protein products of the EGT-derived genes are subsequently synthesized in the cytosol and retargeted to the plastid (Martin et al., 2002; Reyes-Prieto et al., 2006) *via* a sophisticated trafficking system (Li and Chiu, 2010). Some of the cyanobacterial genes also take on functions unrelated to the plastid (Timmis et al., 2004; Kleine et al., 2009). This massive gene relocation process has resulted in mosaic algal nuclear genomes with the cyanobacterium-derived EGT set accounting for 6–20% of the total gene repertoire in Plantae; e.g., glaucophyte *Cyanophora paradoxa* (Reyes-Prieto et al., 2006; Price et al., 2012), extremophilic red alga *Cyanidioschyzon merolae *(Sato et al., 2005; Deusch et al., 2008; Dagan et al., 2013), unicellular green alga *Chlamydomonas reinhardtii *(Deusch et al., 2008; Moustafa and Bhattacharya, 2008), picoplanktonic green alga *Ostreococcus tauri* (Dagan et al., 2013), *Oryza sativa* (Deusch et al., 2008), *Arabidopsis thaliana*, and other land plants (Martin et al., 2002; Deusch et al., 2008; Dagan et al., 2013).

Another source of evolutionary novelty in Plantae is non-cyanobacterial (i.e., Archaea and other bacteria) prokaryote-derived HGT that occurred throughout the history of this supergroup (**Figure 1**). HGT appears to be widespread and is found in all three Plantae phyla; e.g., *Cyanophora paradoxa* (Price et al., 2012), the extremophilic red alga *Galdieria sulphuraria* (Schoenknecht et al., 2013), the mesophilic red alga *Porphyridium purpureum* (Bhattacharya et al., 2013), the red seaweed *Chondrus crispus* (Collen et al., 2013), the green picoprasinophytes *Ostreococcus tauri* (Derelle et al., 2006) and *Micromonas* spp. (Worden et al., 2009), the green algae *Chlorella variabilis NC64A* (Blanc et al., 2010), *Coccomyxa subellipsoidea* (Blanc et al., 2012), *Bathycoccus prasinos* (Moreau et al., 2012), and land plants [e.g., the moss *Physcomitrella patens* (Yue et al., 2012)]. HGT-derived genes have enabled adaptation of red algae to extreme environments (Schoenknecht et al., 2013). A recent genome-wide analysis of *Porphyridium purpureum* showed that ~5% of the gene repertoire in this mesophile was derived from non-cyanobacterial prokaryotes, which is comparable to the number of cyanobacterium-derived EGTs in this genome (Bhattacharya et al., 2013).

A significant source of non-cyanobacterial genes in algal genomes is from the intracellular parasitic bacteria, Chlamydiae (Huang and Gogarten, 2007; Becker et al., 2008; Moustafa et al., 2008; Ball et al., 2013). Many Chlamydiae-derived genes encode proteins with putative plastid functions (Horn, 2008; Moustafa et al., 2008). The results of a recent study suggest that Chlamydiae may once have existed as symbionts in the Plantae ancestor and aided in the harnessing of the cyanobacterial primary endosymbiont (Ball et al., 2013; Baum, 2013). If this hypothesis is true, then many Chlamydiae-derived algal genes could also be considered as examples of EGT from a long-term (now absent) symbiont.

## ENDOSYMBIOTIC GENE TRANSFER OF PLANTAE GENES INTO CHROMALVEOLATES

As described above, like primary endosymbiosis, secondary and tertiary endosymbiosis also led to large-scale gene transfer to the host nuclear genome *via* EGT (**Figure 1**; Lane and Archibald, 2008). This process allows the retention of genes critical for plastid functions because the nucleus of the endosymbiont (e.g., engulfed alga) either shrinks dramatically in size to a nucleomorph (i.e., 500–700 Kbp in cryptophytes; Douglas et al., 2001; Lane et al., 2007; Tanifuji et al., 2011; Moore et al., 2012) and 400 Kbp in *Bigelowiella natans*; Gilson et al., 2006) or is lost outright (Moore and Archibald, 2009; Keeling, 2010). Alga-derived EGT genes have been described in detail from a variety of photosynthetic taxa, including “chromists” (Frommolt et al., 2008), dinoflagellates (Chan et al., 2012b) and *Bigelowiella natans* (Archibald et al., 2003), as well as from ciliates that may once have contained a plastid (Reyes-Prieto et al., 2008).

Whole-genome sequences of photosynthetic chromalveolates and rhizarians provide a global picture of the footprints of algal endosymbiosis. For example, 171 genes with red or/and green algal provenance were identified in the genome of the diatoms *Phaeodactylum tricornutum* (Bowler et al., 2008) and *Thalassiosira pseudonana* (Armbrust et al., 2004). Using more comprehensive methods, thousands of green algal-derived genes were later found in the genomes of these diatoms, which outnumber the contribution from red algae. As described above, this was interpreted as potentially deriving from a cryptic green algal secondary endosymbiosis (added to by independent HGTs) in chromalveolates (Moustafa et al., 2009). Analysis of the genome from the brown, filamentous seaweed *Ectocarpus siliculosus* also revealed a substantial number of green algal-derived (>2000) and red algal-derived (~500) genes (Cock et al., 2010). More than 800 genes with a red algal or cyanobacterial provenance were identified in the genomes of the non-photosynthetic plant pathogens *Phytophthora sojae* and *Phytophthora ramorum* (Tyler et al., 2006), suggesting a photosynthetic past for these taxa [but see (Stiller et al., 2009)]. Recent analyses of complete genome data from the nucleomorph-containing taxa *Guillardia theta* (cryptophyte) and *Bigelowiella natans* (rhizarian), turned up 508 and 353 algal-derived genes, respectively, which account for 7 and 6% of all genes analyzed in these two taxa (Curtis et al., 2012).

From the perspective of algal endosymbiosis, analysis of *Porphyridium purpureum* complete genome data shows that ~40% of its genes are shared with at least one chromalveolate taxon (Bhattacharya et al., 2013). This passage of red algal genes into chromalveolates appears to be very broad in terms of gene function (Bhattacharya et al., 2013). Due to the possible mixotrophic lifestyle of photosynthetic lineages such as *Bigelowiella natans* (Moestrup and Sengco, 2001), the relationship between algal-derived EGT and prey-derived HGT is hard to disentangle. Regardless of the underlying mechanism, Plantae contribution to host genomes of secondary or tertiary endosymbiont-containing algae is significant. These numbers are expected to increase as more Plantae and chromalveolate complete genomes are analyzed.

## RED ALGAE MEDIATE CYANOBACTERIAL GENE TRANSFER INTO CHROMALVEOLATES

**FIGURE 2:**
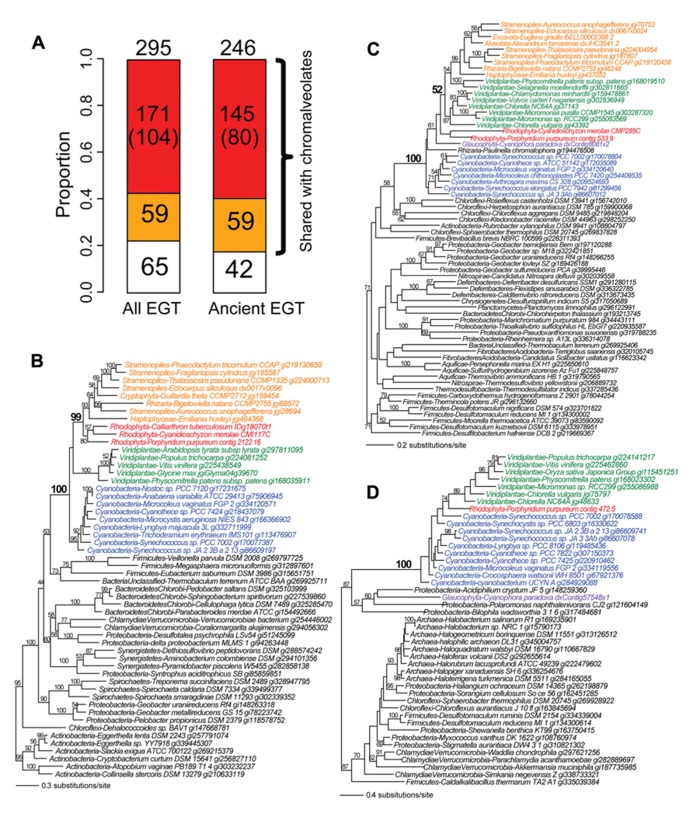
**The fate of cyanobacterium derived EGTs in the red alga *Porphyridium purpureum*. (A)** Proportion of *Porphyridium purpureum* genes shared with chromalveolates. The red color indicates red algal secondary endosymbiotic gene transfer (EGT) reflected by *Porphyridium purpureum*-chromalveolate monophyly. The number in the parenthesis indicates *Porphyridium purpureum*-chromalveolate monophyly with ≥ 60% bootstrap value (e.g., **Figure 2B**). The orange color indicates other scenarios of red/green algal EGT into chromalveolates (e.g., **Figure 2C**). The white color indicates red algal EGT that have no apparent homologs in chromalveolates (e.g., **Figure 2D**). Ancient EGTs refer to genes shared by red algae and glaucophytes or green alga/plants and excludes red algal-specific EGTs. **(B)** Maximum likelihood phylogeny of an ABC transporter. **(C)** Maximum likelihood phylogeny of an acetyl ornithine aminotransferase. **(D)** Maximum likelihood phylogeny of a prenyltransferase. All RAxML bootstrap values were determined using 100 replicates and only bootstrap values ≥50% are shown.

Given the evidence for massive prokaryote-to-eukaryote gene transfer *via* primary endosymbiosis and eukaryote-to-eukaryote gene transfer *via* secondary and tertiary endosymbiosis, we hypothesize that primary plastid-containing algae (red or green algae) have played a central role as mediators of the spread of prokaryotic genes into eukaryotes. We used the phylogenomic results from the recently generated *Porphyridium purpureum* genome (Bhattacharya et al., 2013) to test this idea. Using a cutoff of ≥60% bootstrap support for *Porphyridium purpureum*-cyanobacterium gene monophyly (followed by manual inspection), we identified 295 cyanobacterium-derived (i.e., *via* EGT) genes in the red alga. Of these, 78% (230/295) were shared with chromalveolates (**Figure 2A**) and among these proteins, 74% (171/230) likely owe their origin to red algal secondary endosymbiosis. The latter value was determined by counting all cases of *Porphyridium purpureum*-chromalveolate monophyly, regardless of bootstrap value. When the bootstrap cutoff ≥60% was applied to *Porphyridium purpureum*-chromalveolate monophyly, the number was 45% (104/230). A typical example of this class is an ABC transporter that is shared exclusively by cyanobacteria, red/green algae, and chromalveolates (100% bootstrap value). Among this group, the red alga (including *Porphyridium purpureum*) sequences are monophyletic with chromalveolates (99% bootstrap value, **Figure 2B**). The remaining 59 cases of EGT shared with chromalveolates represent putative outcomes of a cryptic green algal endosymbiosis or have ambiguous evolutionary histories (**Figures 2A, C**, which is a tree of an acetyl ornithine aminotransferase). A total of 22% (65/295) of the 295 EGT-derived genes have no identifiable homologs in chromalveolates (e.g., a prenyltransferase gene tree shown in **Figure 2D**). Because much of EGT presumably took place early in Plantae evolution, similar results are obtained when the analysis is limited to ancient cases of EGT; i.e., genes are counted when shared by *Porphyridium purpureum*, glaucophytes, and/or green algae and land plants (**Figure 2A**).

## RED ALGAE MEDIATE NON-CYANOBACTERIAL GENE TRANSFER INTO CHROMALVEOLATES

**FIGURE 3:**
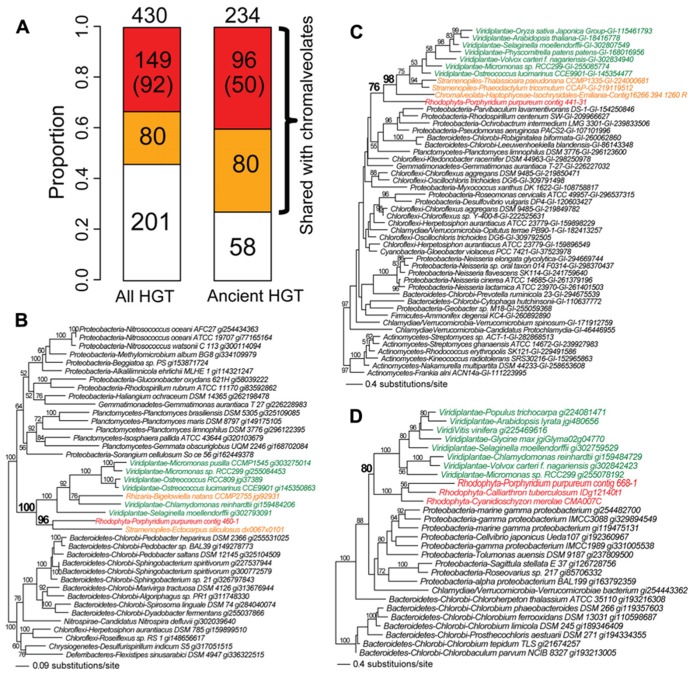
**Fate of non-cyanobacterium derived HGTs in the red alga *Porphyridium purpureum*. (A)** The proportion of *Porphyridium purpureum* HGTs shared with chromalveolates. The red color indicates secondary EGT of HGT-derived genes in red algae based on *Porphyridium purpureum*- chromalveolate monophyly. The number in parenthesis indicates *Porphyridium purpureum*-chromalveolate monophyly with ≥ 60% bootstrap value (e.g., **Figure 3B**). The orange color indicates other scenarios of red/green algal HGTs into chromalveolates (e.g., **Figure 3C**). The white color indicates red algal HGTs that have no homologs in chromalveolates (e.g., **Figure 3D**). Ancient HGTs refer to genes shared by red algae and glaucophytes or green algae/plants, with exclusion of red algal-specific HGTs. **(B)** Maximum likelihood phylogeny of an ABC transporter. **(C)** Maximum likelihood phylogeny of a transmembrane transport protein. **(D)** Maximum likelihood phylogeny of a serine acetyltransferase. All RAxML bootstrap values were determined using 100 replicates and only bootstrap values ≥50% are shown.

We identified the instances of non-cyanobacterium-derived HGT in *Porphyridium purpureum*. This number (following manual inspection) was 430 genes at a bootstrap cutoff ≥60%. Of these, 53% (229/430) is shared with chromalveolates, of which 65% (149/229) is likely derived from red algal secondary endosymbiosis, reflecting *Porphyridium purpureum*-chromalveolate monophyly regardless of bootstrap support (**Figure 3A**). This proportion reduces to 40% (92/229) when the bootstrap cutoff ≥60% is applied to *Porphyridium purpureum*-chromalveolate monophyly (**Figure 3A**). One example is an ABC transporter phylogeny (**Figure 3B**) that includes only bacterial and algal sequences. In this tree, *Porphyridium purpureum* forms a monophyletic group with the brown alga *E. siliculosus* (98% bootstrap value) and is sister to a group of green algae and land plant sequences. The *Bigelowiella natans* sequence is nested within green algae, consistent with a secondary endosymbiotic origin of this gene (**Figure 3B**). The remaining 80 HGT-derived genes shared with chromalveolates represent either cryptic green algal endosymbiosis or ambiguous evolutionary histories (**Figure 3A**). An example is a transmembrane transport protein phylogeny that includes only bacterial and algal sequences. In this tree, green algae and land plants form a monophyletic group with chromalveolates (98% bootstrap value) with the exclusion of *Porphyridium purpureum* (**Figure 3C**). The remainder of non-cyanobacterial HGTs (47%, 201/430) is not shared with chromalveolates (e.g., serine acetyltransferase phylogeny, **Figure 3D**).

Among the 430 cases of non-cyanobacterium HGTs in *Porphyridium purpureum*, 234 are shared with glaucophytes or green algae/land plants and likely represent ancient HGT events, consistent with the prevalence of ancient HGT in Plantae (Huang and Yue, 2013). This is comparable to the number of ancient EGTs (246, **Figure 2A**) derived from the cyanobacterial endosymbiont that are shared by the three Plantae lineages. Because independent HGTs are less likely to result in a large number of shared genes among taxa, the extensive shared footprint of ancient non-cyanobacterial HGT provides additional support for the monophyly of Plantae (Price et al., 2012; Spiegel, 2012). Finally, if we limit our analysis to the 234 cases of ancient HGT (**Figure 3A**), then the proportion of *Porphyridium purpureum* genes shared with chromalveolates increases to 75% (176/234; **Figure 3A**). This approaches the number (83%, 204/246) of ancient EGTs that we identified in our study. These results underline the significance of ancient non-cyanobacterial HGT in enriching red algal genomes and the subsequent movement of these genes *via* secondary endosymbiosis to chromalveolates.

## CONCLUSION

Ancient red algae (e.g., the ancestor of taxa such as *Porphyridium purpureum*) appear to have mediated transfers of ~300 prokaryotic genes into chromalveolates. In addition to the expected transfer of cyanobacterium-derived genes *via* EGT, a comparable number of non-cyanobacterium-derived genes, particularly those acquired early in Plantae evolution, appear to have undergone inter-phylum gene transfer. This role of red algae as mediators of gene transfer (exemplified by *Porphyridium purpureum*) is applicable to endosymbionts of other secondary and tertiary endosymbiosis (e.g., green algae). These data suggest a previously under-appreciated source of reticulate gene ancestry among photosynthetic eukaryotes that has great implications for the origin of novel gene functions in algae and for inference of ancient phylogenetic relationships in the tree of life (Lane and Archibald, 2008; Chan et al., 2012a).

## Conflict of Interest Statement

The authors declare that the research was conducted in the absence of any commercial or financial relationships that could be construed as a potential conflict of interest.
